# Revising the Superorganism: An Organizational Approach to Complex Eusociality

**DOI:** 10.3389/fpsyg.2019.02653

**Published:** 2019-12-03

**Authors:** Mark Canciani, Argyris Arnellos, Alvaro Moreno

**Affiliations:** ^1^Department of Logic and Philosophy of Science, IAS-Research Centre for Life, Mind and Society, University of the Basque Country, Donostia-San Sebastián, Spain; ^2^Complex Systems and Service Design Laboratory, Department of Product and Systems Design Engineering, University of the Aegean, Mytilene, Greece

**Keywords:** eusociality, self-organization, hierarchical regulation, inter-identity, superorganism

## Abstract

Eusociality is broadly defined as: colonies consisting of overlapping generations, cooperative brood care, and a reproductive division of labor where sterile (or non-reproductive) workers help the reproductive members. Colonies of many complex eusocial insect species (e.g., ants, bees, termites) exhibit traits, at the collective level, that are more analogous to biological individuals rather than to groups. Indeed, due to this, colonies of the most complex species are typically a unit of selection, which has led many authors to once again apply the concept of the superorganism to eusocial insects. However, unlike Wheeler, who originally employed the concept from a physiological and evolutionary perspective, today the superorganism is typically understood only from an evolutionary perspective, using group selection. This is because of the widely held view that eusocial colonies are self-organized systems. According to this view, even the most complex eusocial systems can be explained by appealing to a set of local interactions between parts of an initially disordered system (i.e., self-organization), without the need of any hierarchical control. In this paper, we challenge the mainstream view that hierarchical control and regulation does not occur, or is not necessary, in complex eusocial colonies. Using a case study of honey bees (*Apis mellifera*), we develop an alternative to the self-organization approach that focuses on the hierarchical nature of the organization of complex eusocial systems—that we refer to as the *hierarchical-organizational approach*. In addition, we analyze how colonies of eusocial insects show a complex set of interactions between the different organisms that bring forth a new cohesive collective organization, and how in turn the constitutive entities of this collective organization are transformed in this process. This paper argues that an inter-identity (namely the superorganism) emerges at the collective level in complex eusocial colonies, such as honey bees, due to the hierarchically organized network of interactions within the colony.

## Introduction

Eusociality has been at the center of many debates in philosophy and biology for decades because it represents an extremely high form of social integration. It is characterized by colonial groups and broadly defined as: colonies consisting of overlapping generations, cooperative brood care, and a reproductive division of labor where sterile (or non-reproductive) workers help the reproductive members (Wilson and Hölldobler, 2005, p. 13367).

In evolutionary biology, eusociality is commonly seen to be a problem because it raises the issue of how non-reproducing organisms can evolve and persist. It is probably for this reason that, historically, the main focus on the question of eusociality has been on the evolution of eusocial systems, which has implications for various philosophical debates, such as biological altruism, cooperation versus conflict, levels of selection, units of selection, sociality, and more (Hamilton, 1964a,b; Wilson and Sober, 1989; Gadagkar, 1990; Maynard Smith and Szathmáry, 1995; Nowak et al., 2010; Abbot et al., 2011).

However, the maintenance and further evolution from simple to complex eusociality have recently gained more attention (Bourke, 1999; Anderson and McShea, 2001; Hou et al., 2010; Burchill and Moreau, 2016; Fewell and Harrison, 2016) for their implications in other important philosophical debates, such as that of biological individuality, evolution of complexity, self-organization, and more. In particular, the fact that eusocial systems show a high degree of integration raises debates about w*hether eusocial colonies can be considered as biological individuals in their own right rather than just groups*.

In insects, where eusocial organization reaches the highest degree, complex colonies are large with a high degree of polymorphism (a worker caste that is morphologically different from the reproductive caste, as well as, possibly, polymorphism among the worker castes), the loss of reproductive potential and “totipotency” in the worker castes, and complex communication systems (Bourke, 1999; Anderson and McShea, 2001). These complex colonies are, therefore, broadly defined by colony size, degree of polymorphism, worker totipotency, and communication networks. Labeling a eusocial colony “complex” is not just based on arbitrarily chosen parameters, nor is it about just trivial differences among eusocial colonies. There is something objectively different about the intrinsic organization of the complex eusocial colonies compared to simple ones, which is due to the increasing complexity at the colony level^1^ and decreasing complexity at the level of each insect that constitutes the colony (Anderson and McShea, 2001). It has been shown that (as predicted by metabolic scaling theory for unitary organisms) the increase to a larger colony size (and therefore mass) causes lower mass-specific energy use in complex eusocial colonies (Hou et al., 2010; Fewell and Harrison, 2016). Moreover, polymorphism and the loss of worker reproductive potential are associated with large colonies only (Bourke, 1999; Anderson and McShea, 2001). Also, whereas in simple colonies, where the control of colony processes, such as foraging, reproductive division of labor, etc., is typically controlled (almost) solely by the queen, in complex colonies these factors are more distributed so that the overall control is at the level of the colony due to queen-worker and worker-worker interactions (Huang and Robinson, 1992, 1996; Robinson, 1992; Gordon, 1996; Pankiw et al., 1998a,b; Lillico-Ouachour and Abouheif, 2017).

Complex eusocial colonies are, therefore, qualitatively different to simple ones. But how can we explain these differences? It is commonly accepted that the development and maintenance of eusociality in the case of complex eusocial insects (i.e., ants, bees, wasps, and termites) are supported by a complex organization of chemical exchanges^2^. These special chemical substances—called pheromones—act as hormones, but outside the body of the secreting agent, modifying the physiological structure and the behavior of the neighboring members of the colony. Control over other substances could also be employed; for example, royal jelly secreted by honey bee workers, is important for controlling caste determination and the queen’s development.

But there is no agreement in how these chemical mechanisms operate. In fact, the most widely accepted view is that these complex collective patterns are the result of self-organization. According to this view, even the most complex eusocial systems can be explained by appealing to the feedback loops that emerge as a result of a set of local interactions between the parts of the colony, without the need of any hierarchical control (Detrain and Deneubourg, 2006; Hölldobler and Wilson, 2009).

In this paper, we shall argue that this claim cannot be presupposed and must be re-assessed theoretically and empirically. To do so we will provide an alternative, based on a case study, to the current explanations of complex eusociality using a perspective that focuses on the hierarchical nature of the organization of complex eusocial systems. In addition, we will analyze how colonies of eusocial insects show a complex set of interactions between the different organisms that bring forth a new cohesive collective organization, and how in turn the constitutive entities of this collective organization are transformed in this process.

The paper is organized as follows. In section “The Superorganism and Other Explanations of Complex Eusociality,” we will provide a brief historical review and state-of-the-art of explanatory approaches to complex eusociality. In section “The Superorganism,” we review the superorganism theory, highlighting that it was originally employed by Wheeler to argue that colonies are biological individuals in a fuller sense of the term, i.e., both evolutionary and physiological individuals. Whereas today, the concept is typically approached from an evolutionary perspective using multi-level selection and applied to colonies that are units of selection; i.e., denoting evolutionary individuals. The alternative approach—which we will call *organizational*—is today formulated in terms of shallow self-organization. This view holds that colony organization in eusocial insects can be explained in terms of units that interact locally and as a result a global and complex order emerges. Thus, we end this section by reviewing this mainstream approach to explanation of the ontogenetic and physiological aspects of colony organization that we refer to as the self-organization (SO) approach. Then, in section “Honey Bees (*Apis mellifera*): A Case Study of Two Colony Processes,” we will outline our case study of two colony processes in honey bees (*Apis mellifera*). In section “The Hierarchical Organization of Complex Eusocial Colonies,” we will challenge the mainstream view that hierarchical regulation does not occur in the large colonies of complex eusocial insects by developing a different organizational approach, that we will refer to as the *hierarchical-organizational* approach, focusing on the hierarchical organization within complex eusocial colonies as the locus of explanation^3^. Using the case study from section “Honey Bees (*Apis mellifera*): A Case Study of Two Colony Processes,” we will argue that this highly integrated eusocial system is based on a mechanism of regulatory control exerted over the basic level of self-organization processes. Finally, we will compare this example with similar colony processes in other species, showing the key explanatory role played by this hierarchical organization.

## The Superorganism and Other Explanations of Complex Eusociality

In this section, we will give a brief historical review and current state-of-the-art of explanatory approaches to complex eusocial insects.

### The Superorganism

Due to the uniqueness of their organization, eusocial insect colonies have long been thought of as a form of biological individual (Wheeler, 1911, 1928; Emerson, 1939, p. 181). Wheeler (1911) considered eusocial colonies as biological individuals because they act as a cohesive unit; they are individuated and persist over time (once colonies are formed they do not dissolve or merge with other colonies); they undergo development (as opposed to being formed by the aggregation of a group of solitary insects); and most importantly, because of the reproductive division of labor, colonies of some species are the reproducing unit. This led Wheeler (1920, p. 117, 1928, pp. 23–24, 304–305) to apply the concept of the “superorganism” to eusocial insect colonies. Emerson (1939), inspired by the earlier work of Wheeler, also applied the superorganism concept to eusociality. However, for Emerson (1952), the superorganism concept was primarily a tool for analogical reasoning. He argued that focusing on the analogies (and dissimilarities) between eusocial colonies and organisms can guide eusociality researchers to discover the processes and integrating mechanisms that enable the emergence of biological individuality at the level of the colony. Around the 1960s, there was an increasing preference among eusociality researchers for more reductive approaches, due to the gene-centered perspective of the Modern Synthesis (Wilson, 1971). As a result of Emerson’s analogical notion of the concept, and the preference for more reductive approaches such as kin selection, the superorganism saw a radical decline after the 1960s (Wilson, 1971; Wilson and Sober, 1989).

Wilson and Sober (1989) argued for a revival of the superorganism concept, but based on an evolutionary notion of biological individuality^4^. For Wilson and Sober, the defining feature of organisms, and thus superorganisms, is the ability to directly partake in natural selection. Or in other words, what separates organisms from other biological systems/groups is that they are units of selection: “Individuals acquire the exquisite functional organization that justifies their status as organisms by the process of natural selection” (Wilson and Sober, 1989, p. 339). Wilson and Sober argued that eusocial colonies, as well as other groups of organisms, also exhibit functional organization, and thus should be considered as higher level organisms (superorganisms).

In order to extend their definition of organism to eusocial colonies, Wilson and Sober (1989) relied on the notion of multi-level selection. Multi-level selection (MLS) theory argues that selection can operate at multiple levels simultaneously, i.e., at the levels of the gene, cell, multicellular organism, group, population, etc. MLS can be used to track the effects of group-living on individual fitness (MLS1) or, importantly, to argue for group selection (MLS2) (Damuth and Heisler, 1988). By focusing on the ratio of within-group and between-group selection, authors can determine if selection primarily acts at the individual level (MLS1) or at the group level (MLS2) for a specific (group-structured) population (Hölldobler and Wilson, 2009; Hamilton and Fewell, 2013). Indeed, Wilson and Sober argued that eusocial colonies can be considered as superorganisms if they achieve a high degree of internal cooperation (functional organization) such that *between-colony* selection is greater than *within-colony* selection. Or in other words, if colonies qualify as units of selection, then they are superorganisms.

Although the superorganism theory went through a sharp decline for almost two decades, it is once again at the forefront of eusociality research. Today, authors use MLS to show that, for many eusocial insect species, colonies are the unit of selection, i.e., evolutionary individuals (Hölldobler and Wilson, 2009; Queller and Strassmann, 2009; Okasha, 2014; Helanterä, 2016). However, while the evolutionary aspects of eusocial insect colonies are important (e.g., the transition from the selection of reproductives to the selection of colonies) the physiological/ontogenetic aspects are equally important. For example, the relations and interactions between the members of the colony are important to understand the proximate causes for the functional integration that enables colony selection (see Arnellos et al., 2014, for an analogous argument for multicellular individuality). “The challenge is to understand the complex mechanisms that enable a colony to function *as a single organism*, exactly as imagined by Wheeler so long ago” (Wilson and Wilson, 2007, p. 342, emphasis in original).

Despite this, the superorganism is rarely approached from a physiological perspective today, as it was originally done so by Wheeler. This is because of the mainstream view that hierarchical regulation does not occur, or indeed is not necessary, in the large colonies of the more complex eusocial species (Boomsma and Franks, 2006). Arguments are made along the following lines, “[…] their colony as a whole lacks command and control by a still higher-level system. It therefore must be self-organized” (Hölldobler and Wilson, 2009, p. 58). In other words, because colonies lack physical contiguity and any type of organ or nervous system at the collective level (i.e., colony level), such as in multicellular organisms, it is argued that top-down hierarchical control does not occur. Consequently, current explanatory approaches that focus on the physiological and proximate causes for colony cohesiveness (i.e., the actual organization) are centered around the concept of self-organization—which we will refer to as the self-organization (SO) approach—(Boomsma and Franks, 2006; Detrain and Deneubourg, 2006; Fewell et al., 2009). Consequently, even colonies of the most complex eusocial species (with polymorphic castes, complex division of labor, colony selection, etc.) are typically conceived of as *self-organized groups*.

### The Self-Organization Approach

In the SO approach the concept of self-organization, developed in thermodynamics to explain spontaneous macroscopic patterns emerging in physical and chemical systems from the interactions of their microscopic parts, is applied to eusocial insect colonies^5^ in an attempt to explain colony organization. This approach began around the 1980/90s and was developed by Bonabeau, Deneubourg, Theraulaz, and Franks, among others (Bonabeau et al., 1997; Boomsma and Franks, 2006; Detrain and Deneubourg, 2006; Fewell et al., 2009). The main tenet of SO theories is that complex colony level phenomena can occur in eusocial insect colonies without a hierarchical organization and control, instead they are the result of a flat network of locally distributed interactions among the parts (in this case the individual insects).

In the context of eusocial systems, self-organization is defined as positive and negative feedback loops resulting from multiple interactions between the insects, and the amplification of random fluctuations in those interactions (for more details, see Bonabeau et al., 1997). For example, in the ant genus *Pheidole*, it has been shown that in some species the colony can respond to substandard caste ratios *via* feedback loops, reverting caste ratios to optimum levels over a few worker generations (Lillico-Ouachour and Abouheif, 2017). If the ratio of minor workers to soldiers is too skewed in favor of soldiers, the increased number of soldiers will inhibit further soldier development in the larvae *via* a negative feedback loop. The soldiers give off a pheromone that inhibits larvae developing into soldiers; therefore, if soldiers are present then more larvae will develop into workers than soldiers, or conversely, if there are too few soldiers, then this will increase soldier development in larvae due to the removal of the inhibitory effect of the soldier’s pheromone. Thus, through the soldier’s pheromone negative feedback loop, the minor worker-to-soldier ratio is maintained at an optimum level for the colony.

Since the “elementary” units that make up complex eusocial insect colonies are complex agents, in this context, self-organization is sometimes combined with the concept of stigmergy and referred to as stigmergic self-organization (Bonabeau et al., 1997; Holland and Melhuish, 1999). It has been shown that through very simple behavioral rules (or interpretative decision making), complex colony level processes can occur *via* self-organization. For example, Holland and Melhuish (1999) found that robots programmed with a few simple response rules could sort two distinct types of frisbee in a given space, and put one type into a cluster. The robots achieved this by responding differently to different stimuli, for example; all frisbees that are not in contact with another frisbee are picked up, if the robots encountered ringed frisbees that were in contact with any other frisbee, then they cannot pick them up and move them, but the plain type of frisbee is always picked up and moved when encountered. After several hours, this results in a cluster containing mainly ringed frisbee. The process of clustering different types of objects occurs in certain eusocial colonies, for example brood sorting in some ant species (Holland and Melhuish, 1999).

Another particularly interesting approach under the general SO approach is that of the so-called “response threshold theory” (Robinson, 1992; Page and Erber, 2002). The response threshold theory suggests that some individuals will have lower response thresholds for some tasks, say pollen foraging, and will react first to any stimulus for this behavior (reduced pollen stores). As they undertake this behavior, the stimulus is reduced and those individuals with a higher threshold for this behavior do not respond, thus only a subset of the group typically responds. At the same time, those that did not respond to pollen foraging may have lower response thresholds for other tasks, like water foraging, and so on. So, the response threshold theory predicts that division of labor will occur within groups due to the natural variation in stimulus thresholds of the individuals, which has also been experimentally verified (Page and Erber, 2002). Applied to complex eusociality, this theory suggests that response thresholds may be correlated with physiological or temporal castes and, thus, division of labor in complex eusociality is an example of natural selection stabilizing patterns of variation in response thresholds (Robinson, 1992; Page and Erber, 2002; Schulz et al., 2002). In other words, the response threshold theory provides a good explanation of how certain variation among the parts leads to the propensity for self-organization in groups, i.e., general division of labor.

As above, in the SO approach, the colony level phenomena are explained as being just the result of local interactions that together bring forth a global order among the insects. Hence, this global order is not due to any top-down control, but spontaneously emerges from the local interactions of the agents (Boomsma and Franks, 2006; Detrain and Deneubourg, 2006). Therefore, the mainstream view is that hierarchical control and regulation does not occur in complex eusocial colonies.

In the next sections, we will argue that this mainstream view is unjustified, and that research into hierarchical regulation in the complex species should not be neglected, as has been the case under the SO approach. We develop an alternative organizational approach (the *hierarchical-organizational* approach) that assesses if there is hierarchical organization within complex colonies, which “modulates” (i.e., regulates and controls on) the self-organized dynamics within the colony system; i.e., this approach will be able to assess if colony organization is the result of self-organization only or *also* and *mainly* due to hierarchical regulation and control. Consequently, this approach would be better suited to assess the issue of whether complex eusocial insect colonies should be considered biological individuals or not. This is because, if there are colonies with hierarchical organization, then an argument can be made that the colony is in “control” rather than the insects that instantiate it, i.e., the colony organization is not solely the result of self-organization but a higher level organization that exerts top-down control on its parts.

## Honey Bees (*Apis Mellifera*): A Case Study of two Colony Processes

Honey bees (*Apis mellifera*) are a well-studied complex eusocial species; they have large colonies, typically tens of thousands of bees (Smith et al., 2016), with polymorphism between the reproductive and worker castes (Lyko et al., 2010), and workers that have low reproductive potential (Maisonnasse et al., 2010b; Ronai et al., 2015). Here we will explore in detail two examples of colony level processes that are essential to colony development and maintenance: temporal polyethism/worker castes and queen/worker production. However, these are just a sample of the total set of processes that occur at the colony level, we only focus on these particular two due to restrictions of space in this article.

### Temporal Polyethism and Worker Castes

As with all other eusocial species, honey bees exhibit a reproductive division of labor (Lyko et al., 2010; Ronai et al., 2015); the queen is the only reproductive member and workers are not reproductively active. Additionally, *A. mellifera* also exhibit a further division of labor among the workers (Johnson, 2008); workers undergo a temporal polyethism schedule and, consequently, within colonies there are temporal worker castes. The worker castes of *A. mellifera* are the following:

1. *Nurses*: specialize in feeding and attending the brood, as well as feeding the queen and other members of the colony. They have low juvenile hormone (JH) and high vitellogenin (Vg) levels, and large hypopharyngeal glands that are used to produce jelly to feed other colony members;
2. *Nest workers*: specialize in other intranidal (inside the nest) tasks, such as comb construction and maintenance, ventilation, receiving nectar and processing it into honey, storing honey and pollen, and more. They have increasing JH and decreasing Vg levels, and medium-sized hypopharyngeal glands that start producing enzymes for processing nectar into honey instead of producing jelly;
3. *Foragers*: specialize in extranidal (outside the nest) tasks, such as foraging for nectar, pollen, and water. They have the highest JH levels and lowest Vg levels, and small inactive hypopharyngeal glands (Seeley, 1982; Johnson, 2008).

Adult worker bees transition through the different castes as they age. Typically, during the active months (spring to autumn) of honey bee colonies, the temporal polyethism schedule is the following: workers are *nurses* from around 2–11 days old, *nest workers* 11–18 days old, and *foragers* around 18+ days old (Johnson, 2008).

Levels of JH and the glycolipoprotein Vg, which are biosynthesized by each bee, have been shown to play integral roles in the temporal polyethism schedule among the worker bees (Amdam and Omholt, 2003; Hölldobler and Wilson, 2009). JH biosynthesis in workers causes further physiological development, inducing the change from intranidal to extranidal workers, and Vg biosynthesis has the reverse effect, inhibiting the physiological development of workers (Nelson et al., 2007). Increasing JH levels causes the hypopharyngeal glands to start producing enzymes for nectar processing instead of being able to produce jelly for brood food or royal jelly (a process which relies on Vg); it also causes the further development of flight muscles, and causes an increase in the biosynthesis of biogenic amines. Additionally, Vg has been shown to influence foraging preference (pollen/nectar) and even the lifespan of workers (Amdam and Omholt, 2003; Nelson et al., 2007).

**Figure 1:**
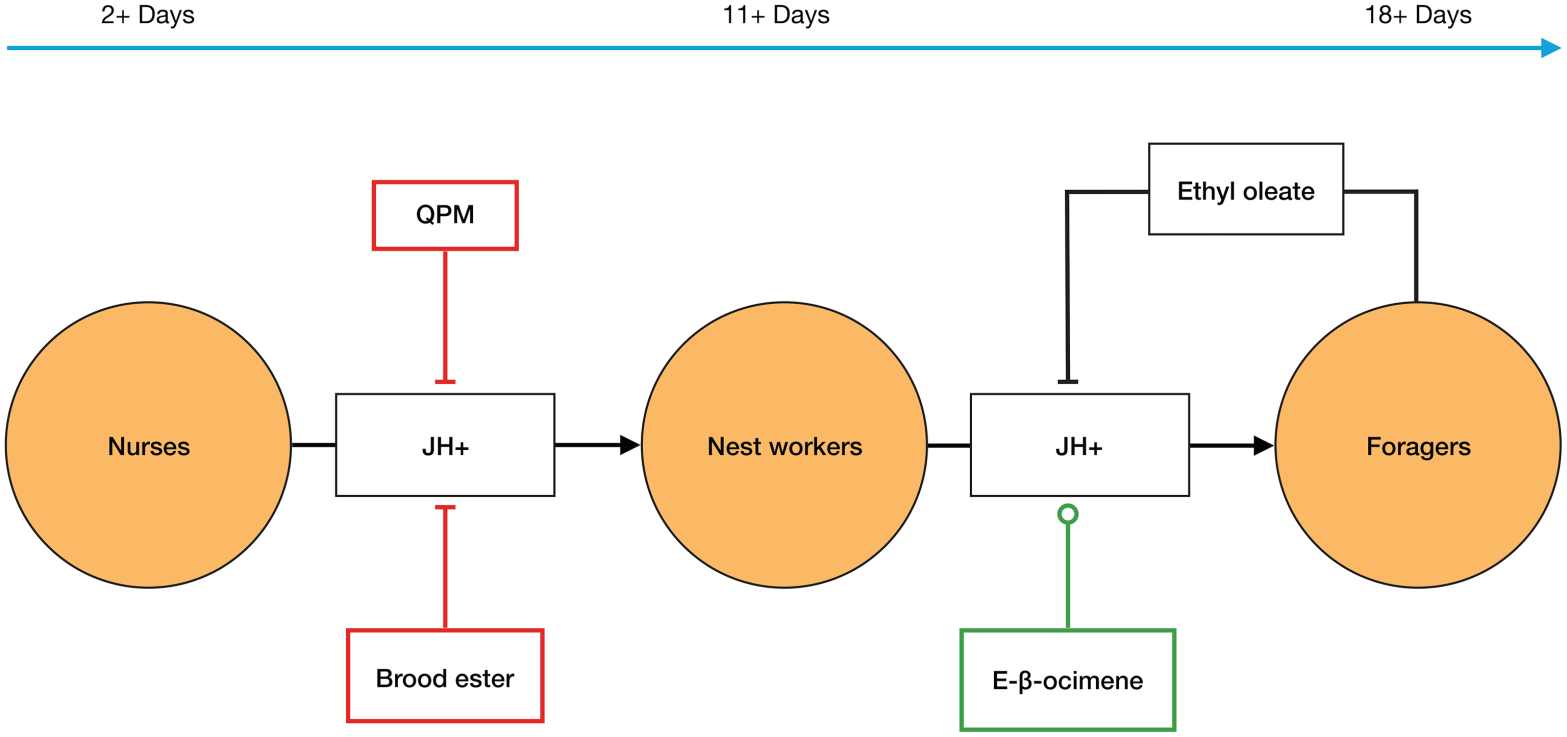
Temporal polyethism. Worker bees, in *A. mellifera*, transition between the temporal worker castes, *orange circles*, as internal levels of juvenile hormone (*JH*) increase, *represented as JH+*. JH biosynthesis is affected by external factors, including inter-member signals: *QPM, brood ester, E-β-ocimene,* and *ethyl oleate*. QPM, brood ester, and ethyl oleate inhibit JH biosynthesis, *flat-ended lines*, and E-β-ocimene promotes JH biosynthesis, *circle-ended line*. The inter-member signals allow the ratio of the temporal worker castes to be controlled at the colony level, *see text for more details*. The timeline represents the typical age of workers in each caste in the active summer period.

In addition to the internal elements JH and Vg, signals from the queen, brood, and the forager caste also affect the temporal polyethism schedule in each worker, and therefore also the ratio of workers within each caste (Figure 1). *Firstly*, the queen has an inhibitory effect on JH biosynthesis in workers *via* her pheromone mix (QPM)^6^. It has been demonstrated that, in the presence of QPM, workers have significantly delayed JH biosynthesis compared to those not exposed to QPM (Kaatz et al., 1992; Pankiw et al., 1998a). *Secondly*, the brood releases signals that affect the polyethism schedule of workers. Young brood emits *E-β-ocimene*—a volatile pheromone that is transmitted into the nest aerially—that appears to target nest workers and causes increased development in them so that they transition to foragers sooner, probably by increasing JH biosynthesis. However, older brood emits *brood ester* pheromone, which is transmitted on contact, that targets nurses, delaying their development most likely by inhibiting the biosynthesis of JH (Maisonnasse et al., 2010b). *Finally*, the forager caste releases a signal that affects the temporal polyethism schedule of younger workers. Foragers produce *ethyl oleate*^7^ that gets transmitted *via* trophallaxis when foragers pass their nectar loads to nest workers, who deposit nectar in the comb. It has been demonstrated that ethyl oleate inhibits the nest workers from transitioning into the forager caste by slowing down their development (Leoncini et al., 2004).

### Queen/Worker Production (Reproductive Caste Determination)

**Figure 2:**
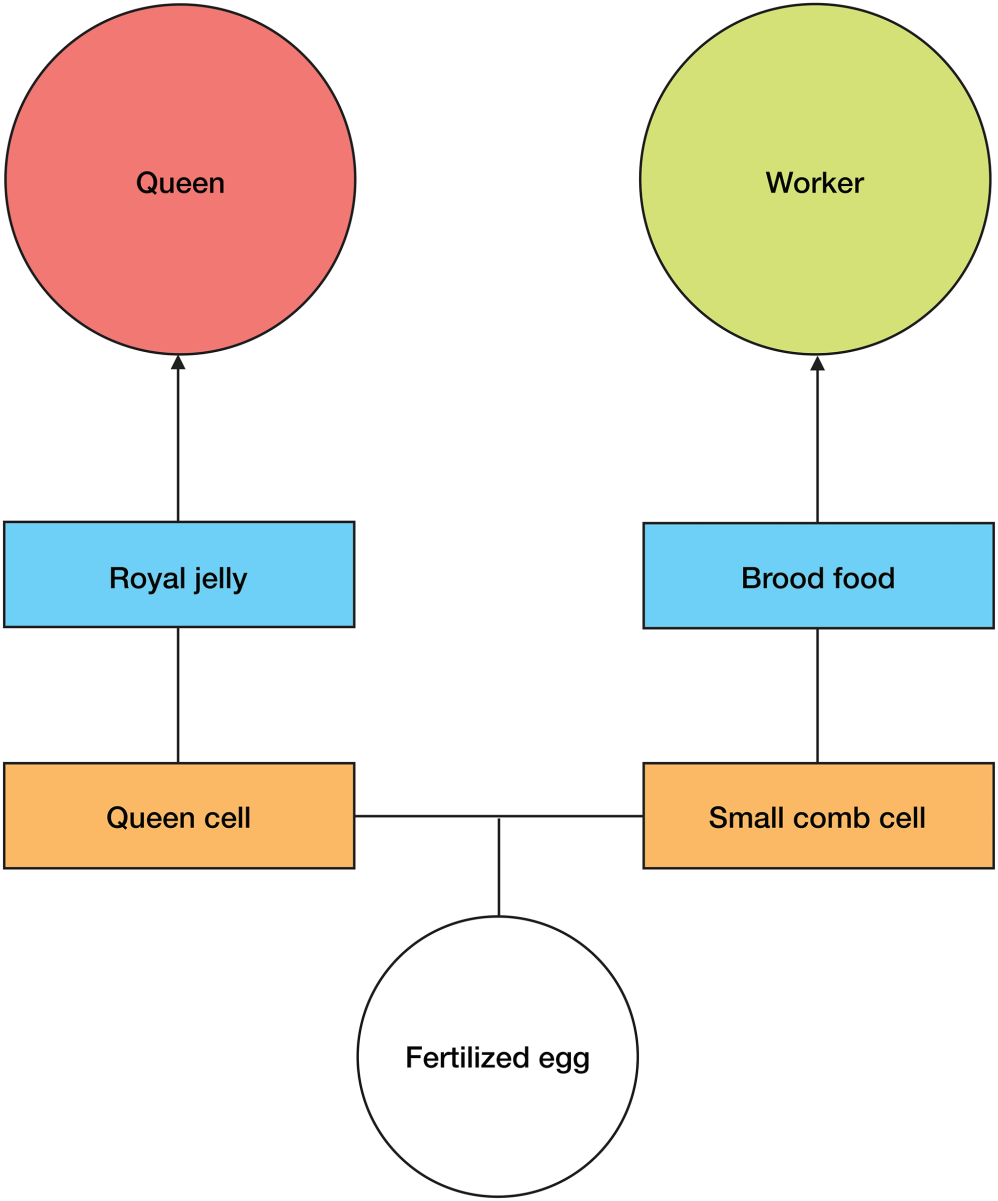
Queen/worker production. Any fertilized egg can develop into a queen or worker in *A. mellifera*. The type of comb cell, *orange squares*, and diet, *blue squares*, determine the development of larvae. Large queen cells and royal jelly cause larvae from fertilized eggs to develop into queens, whereas small comb cells and brood food cause equivalent larvae to develop into workers. Royal jelly induces an epigenetic change within the larvae. The size of the comb cell constrains larval growth. *See text for more details.*

In *A. mellifera*, reproductive/worker caste determination is not genetically predetermined, i.e., any fertilized egg can be used to produce a queen or a worker (Wang et al., 2015). There are, consequently, signals and mechanisms present in the brood stage that determine the development of a particular larva into either a queen or worker (Figure 2). Queen-brood require large vertically-protruding comb cells, which are produced by nest workers. This is because in the larval and pupal stages, queen-brood are much larger than worker-brood, and so the increased volume of the larger comb cells is essential for their proper growth (Wang et al., 2015).

However, it is not only the size of the comb cell that determines the development of fertilized eggs into queens or workers, the type of diet that the brood receives in the larval stages is also an important factor. Nurse workers will begin feeding the larvae as soon as they hatch (eggs hatch after 3 days); worker-larvae receive “brood food”—which is a mixture of jelly, honey, and pollen—while queen-larvae receive a specialized diet of royal jelly and pollen (Beetsma, 1979; Wang et al., 2015). This is significant because it has been experimentally shown that newly hatched larvae (from fertilized eggs) that are fed a diet exclusively of royal jelly during the larval stage will develop into queens, whereas larvae fed with brood food will develop into workers (Page and Peng, 2001; Wang et al., 2015). Lyko et al. (2010) found that it is not just the higher nutritional value of the royal jelly that affects the larvae development. As well as accelerating metabolism and increasing growth, elements of the royal jelly (most likely phenyl butyrate) affect DNA methylation in the developing larvae by silencing DNA methyltransferase 3. Royal jelly, therefore, induces an epigenetic change in the developing larvae.

## The Hierarchical Organization of Complex Eusocial Colonies

We will now highlight the key organizational aspects for the regulation of the two colony level developmental processes—temporal polyethism/worker castes and queen/worker production—described above for *A. mellifera,* and compare them, when applicable, to equivalent processes in *V. vulgaris* and the ants *Pheidole*. We do this in order to emphasize that there are key organizational differences between colonies of different eusocial insect species. We will then argue that in at least some complex eusocial insect species, like *A. mellifera* and possibly more, colonies exhibit a form of hierarchical organization, which exerts a top-down control on the development of its members. In both examples, inter-member signals are crucial for the colony level process, which we have summarized in Tables 1, 2. We start by discussing temporal polyethism.

**Table 1:** Temporal polyethism and caste ratio control in *A. mellifera.*

	Signal	Source	Target	Role
First-order signals	JH	Internal to each worker	Internal to each worker	Increased JH levels induce worker development to next worker caste
	Vg	Internal to each worker	Internal to each worker	High Vg levels are required for nursing, additionally high Vg levels inhibit JH biosynthesis
	Ethyl oleate	Foragers	Nest workers	Inhibits nest workers transitioning to the forager caste
Second-order signals	QPM	Queen	Nurses and nest workers	Inhibits JH biosynthesis and thus worker development
	Brood ester	Old brood	Nurses	Inhibits JH biosynthesis and thus worker development
	E-β-ocimene	Young brood	Nest workers	Induces worker development (nest worker to forager), possibly by inducing JH biosynthesis or inhibiting Vg biosynthesis

**Table 2:** Queen/worker production in *A. mellifera.*

	Signal	Source	Target	Role
First-order signals	Internal developmental constraints of larvae	Internal to each larva	Internal to each larva	Internally controls the development of larvae
Second-order signals	Queen cells	Nest workers	Queen-larvae	Larger comb cell volume allows for the increased growth of queen-larvae
	Worker cells	Nest workers	Worker-larvae	Smaller comb cell restricts growth of worker-larvae
	Royal jelly	Nurses	Queen-larvae	Induces the queen developmental program by causing an epigenetic change
	Brood food	Nurses	Worker-larvae	Induces the worker developmental program by not causing an epigenetic change

### Regulating Temporal Polyethism and Worker Castes

It has been suggested that JH and Vg form a regulatory network within each worker bee since they mutually inhibit one another (Amdam and Omholt, 2003). Typically, levels of Vg are high after first eclosion and naturally decrease with age. High levels of Vg delay the biosynthesis of JH in young adult workers. As Vg decreases, the increasing JH levels inhibit further Vg expression (Nelson et al., 2007). Their mutual inhibition and purely intra-organismal action render JH and Vg dynamically *coupled* to the internal development system of each worker bee. It is in this sense that they are considered to act as *first-order* signals on the temporal polyethism schedule within each worker, and by extension on colony development also.

Added to this, the inter-member signal *ethyl oleate* from the forager caste can also be considered as the same type of signal. This is because the inhibitory effect of ethyl oleate on the nest worker caste is due to a straightforward negative feedback loop, i.e., the presence of foragers inhibits the development of nest workers into the forager caste, but the absence of foragers allows nest workers to develop into foragers. Moreover, workers only begin to produce ethyl oleate at significant enough levels to act as a signal when they reach the forager caste (Leoncini et al., 2004). Thus, the operation of ethyl oleate on this developmental process is tightly coupled to the internal constraints of the individual workers, i.e., there must be older workers (foragers) present in order for ethyl oleate to act as an inhibitory signal on younger workers’ (nest workers) development.

However, the inter-member signals *QPM*, *brood ester*, and *E-β-ocimene* seem to act as different types of constraints on the temporal polyethism schedule. This is mainly because, based on the operation of these signals, they can be considered as dynamically decoupled from the systems that they modulate, i.e., the internal developmental systems of individual workers. All these signals affect the temporal polyethism schedule of workers—QPM and brood ester slow worker development, and E-β-ocimene induces worker development. And although these signals work in concert with the internal developmental systems of the workers, they operate at different timescales than the internal signals of JH and Vg. For instance, the queen is, typically, a constant presence in the colony and therefore the presence of QPM and its influence on the temporal polyethism schedule of the workers are not reliant on a change in concentration of the workers and/or on a direct feedback loop, as is the case with forager-derived ethyl oleate. The same is true of brood ester and E-β-ocimene, since they derive from the brood, which are not a part of the temporal polyethism or the worker castes.

The presence of such *second-order* signals (QPM, brood ester, and E-β-ocimene) on the temporal polyethism of the workers allows for the hierarchical control of the worker caste ratio. This is because the control of the ratio of workers within each caste is not solely reliant on the self-organization of the workers themselves; i.e., it is not solely reliant on a signal (ethyl oleate) from the foragers inhibiting the development of nest workers *via* a negative feedback loop. Instead, additional to the self-organization dynamics of the workers, there are different types of signals (QPM, brood ester, and E-β-ocimene) that modulate the development and temporal polyethism schedule within each worker (Table 1). In other words, *the control and regulation of the ratio of workers within each caste is not dependent on a change in concentration of the workers themselves (i.e., the growth of new workers) but instead on second-order signals that can modulate the existing workers*. For example, when a colony reproduces *via* swarming, the queen and a large proportion of the workers, from all castes (i.e., of different ages), will leave the old nest in order to establish a new nest site (Smith et al., 2016). However, since it takes at least 3 weeks for the colony to produce new workers after it establishes a new nest, the current worker population will be predominately formed of older workers, i.e., the majority of the workers will be older than is typical for the nurse caste (Robinson et al., 1989; Smith et al., 2016). It has been shown that old workers (even those that have been foragers) can revert back to the nurse caste, with these “reverted” nurses even having low JH levels and regenerated hypopharyngeal glands in order to feed the brood (Robinson, 1992). This could be due to the presence of second-order signals. More specifically, older workers can revert to the nurse caste due to *QPM* and *brood ester* inhibiting JH biosynthesis, and consequently allowing Vg biosynthesis and the reactivation of the hypopharyngeal glands. Additionally, *E-β-ocimene* would counteract this by promoting JH biosynthesis to ensure that not too many workers revert to nurses and, thus, ensuring an equal balance between the worker castes. Thus, the plasticity of the temporal polyethism is controlled by the second-order signals that act on the internal development systems of the workers, consequently allowing *the whole colony* to regulate the ratio of workers in each caste.

But this is not the case for all eusocial insect species. For example, in *Pheidole* ants, soldier/minor worker caste determination does not appear to involve second-order signals. *Pheidole* ants do not exhibit temporal castes but many exhibit physical worker castes, typically minor worker and soldier castes (Lillico-Ouachour and Abouheif, 2017). As discussed above, soldier pheromones from adult soldiers present in the colony inhibit worker-larvae from developing into soldiers. However, we suggest that, similar to the operation of the ethyl oleate in *A. mellifera*, the inhibitory effect of the soldier pheromone is also dependent on a change in the concentration of the soldiers themselves; hence, its action on worker-larvae development is dynamically *coupled* to worker-larvae development; i.e., the activation of the inhibitory effect of the soldier pheromone relies on the growth of new soldiers. Thus, the soldier pheromone can be considered as a first-order signal on soldier/minor worker caste determination. The soldier pheromone does allow for the ratio of the morphological worker castes (soldiers and minor workers) to be controlled collectively in *Pheidole*. But this type of collective control is localized in the soldier caste themselves *via* the negative feedback effect of the soldier pheromone.

### Regulating Reproductive/Worker Caste Determination

Control of the temporal polyethism schedule of workers is important for *A. mellifera* colonies because the presence of worker castes allows for further second-order signals. This is clear in the case of caste determination. As we have shown above, the development of a female into either a queen or worker is determined in the larval stages by two factors: the type of comb cell and type of diet. These factors derive from the worker castes; the comb is built by the *nest worker* caste and larvae are fed by the *nurse* caste. Importantly, because the worker castes are continually maintained by the colony (*via* second-order signals—see above), the nurse and nest worker castes can produce their respective signals on caste determination within larvae when required. In other words, the production of these signals is not reliant on a feedback mechanism or change in concentration of the workers, instead they can be produced by the (perennial) nurse and nest worker castes when required by the colony. Specifically, the nest worker caste will produce *worker cells* when the colony requires workers or produce *queen cells* when the colony requires queens (either in the reproductive stage or to replace the old queen). The nurse caste feeds all larvae present in the colony, they feed *brood food* to larvae in worker cells and *royal jelly* to larvae in queen cells. It can thus be argued that the nurse and nest worker castes are dynamically decoupled from the process of caste determination. In other words, the nurse and nest worker castes operate at a different timescale to the systems that they modulate, i.e., the internal developmental systems of the larvae. Therefore, the type of cell and type of diet can be considered as second-order signals on queen/worker caste determination (Table 2). This enables caste determination to be hierarchically controlled at the collective level, rather than being regulated locally *via* self-organization. To further illustrate this point, it will be useful to briefly compare this to a case of caste determination in another species, namely the common wasp (*Vespula vulgaris*).

Things are different in the queen production process in the common wasp (*V. vulgaris*), a species which can be considered at the center of the eusociality complexity spectrum (Bourke, 1999). In wasp colonies, there are no worker castes but there is a form of polymorphism between the queen and workers; queens are larger than workers (Potter, 1964; Jeanne, 1980). Similar to *A. mellifera*, any fertilized egg can develop into a queen or worker in *V. vulgaris*. Thus again, there are mechanisms in the brood stage that determine the development of larvae from fertilized eggs, namely, the size of the comb cell and the amount of nutrition. Increased nutrition causes queen-larvae to grow larger than worker-larvae, which is necessary for the production of queens, and the larger comb cells allow space for this increased growth (Archer, 1972). The comb is constructed, *via* stigmergic self-organization, by all workers—due to the lack of worker castes—but the production of large *reproductive*-comb is determined by a change to the inter-member signal QPM. The QPM of older queens induces the production of reproductive-comb by the workers (for more details, see Potter, 1964). There is no specialized diet for queen-larvae in *V. vulgaris,* but the frequency and quantity of food differ between different layers of the comb. Broods in comb cells that are closer to the nest entrance are fed first and most frequently by returning foragers (Archer, 1972). Reproductive-comb are the last comb layers to be produced by the colony (in the reproductive stage with mature queens) and they are the closest to the nest entrance (Potter, 1964). Moreover, nutrition levels naturally increase as the colony matures (Archer, 1972). As a colony increases in size, there are more workers present that can forage for food, and the resultant increase in foraging causes worker-brood to grow larger. Consequently, the colony also produces larger workers as it matures, which live longer and can collect more food when foraging compared to smaller workers (Richards, 1971; Archer, 1972). Therefore, queen-larvae receive more food compared to worker-larvae and grow larger due to the large comb cells.

The increased nutrition that *V. vulgaris* queen-larvae receive is ultimately reliant on self-organization—the presence of larger workers in the mature stages of the colony has a positive feedback effect on colony nutrition levels. However, the large comb cells required by queen-larvae are determined by a second-order signal (QPM) but in the separate earlier process of comb construction. There are, therefore, second-order signals involved in queen production in *V. vulgaris* but to a lesser degree than in *A. mellifera*. It can be argued that in *V. vulgaris*, queen production is practically determined by the queen—the state of the QPM determines the production of reproductive-comb, and, even though nutrition levels increase due to self-organization, worker foraging is induced by the presence of the queen (Potter, 1964, p. 50).

Instead, in *A. mellifera* the production of queens is determined by the higher order collective organization. Royal jelly, which causes an epigenetic change in queen-larvae, acts as a second-order signal; it is independent of the internal development systems of the developing larvae that it modulates. Also, this second-order signal derives from the *nurse* temporal caste (rather than from the queen). Moreover, the production of reproductive-comb is not dependent on the QPM in the same way as it is in *V. vulgaris*. In *V. vulgaris,* comb construction ceases in queenless colonies (Potter, 1964), whereas in *A. mellifera* the nest workers will still construct comb, in this case particularly reproductive-comb, if the queen dies (Maisonnasse et al., 2010a). Thus, in *A. mellifera*, there are second-order signals that are essential to the process of queen production, but they derive not only from the queen but also from the temporal worker castes. In other words, queen production is also modulated by other parts/sections of the colony (nurses and nest workers) that are dynamically independent from the process of reproductive caste determination. Therefore, the development of fertilized eggs into either workers or queens in honey bees is determined much more globally than in the case of *V. vulgaris,* i.e., at the level of the whole colony.

In can be argued, consequently, that in *A. mellifera*, the network of interactions forms a complex higher order organization that is dynamically decoupled from the operation of the lower level parts (the bees) and which determines (or is in “control” of) the development of the colony. Whereas in other, less complex species, such as *V. vulgaris*, the higher order organization is more basic and coupled to the operation of the lower level parts, specifically the queen.

## Conclusions

Colonies of many complex eusocial insect species exhibit traits, at the collective level, that are more analogous to biological individuals rather than to groups (Anderson and McShea, 2001; Hölldobler and Wilson, 2009). For example, the mass-specific energy use in the large colonies of complex species is similar to that of individual organisms (Hou et al., 2010). Moreover, polymorphic and behavioral worker castes, which enable more complex division of labor, only occur in colonies of the more complex species (Bourke, 1999). Indeed, due to this, colonies of the most complex species are typically the unit of selection, which has led many authors to once again apply the concept of the superorganism to eusocial insects (Hölldobler and Wilson, 2009; Haber, 2013). However, unlike Wheeler (1928), who employed the concept from a physiological and evolutionary perspective, today the superorganism is typically understood only from an evolutionary perspective, using MLS. This is because of the mainstream view that hierarchical control does not occur in the large colonies of complex eusocial insects, which led to the prevalence of the SO approach. While the SO approach has been very insightful in the recent decades, particularly with regard to the explanation of many collective phenomena in eusocial insects, we have argued that this approach may not be fully adequate for all species. This is because hierarchical organization can occur in the more complex species.

Thus, in this paper we challenged the idea that hierarchical regulation does not occur, or is not necessary, in the large colonies of complex eusocial insect species. We did so by developing the *hierarchical-organizational* approach, using the case study of *A. mellifera*. From the assessment of the colony processes, discussed in section “The Hierarchical Organization of Complex Eusocial Colonies,” we argued that colonies of *A. mellifera* are not solely the result of self-organization, but instead exhibit a hierarchical organization.

In *A. mellifera*, not only is there a physiological specialization for reproduction (the queen) and “metabolism” (worker castes), there is also a structured division of labor among the worker castes, based on the temporal polyethism, which is characterized by physiological and behavioral differences among each caste. For example, the state of the hypopharyngeal glands differs in each temporal caste; *nurses* have the largest hypopharyngeal glands that they use to produce jelly for inter-member feeding, *nest workers* have mid-sized glands that they use to process nectar into honey, and *foragers* have the smallest glands that are inactive. We argued that the temporal polyethism schedule within each worker is regulated hierarchically at the colony level *via* second-order signals (Table 1). Substances from the queen (QPM) and the brood (brood ester and E-β-ocimene) act as second-order signals on the internal developmental system of the workers (i.e., JH and Vg biosynthesis) allowing the ratio of workers in each caste to be hierarchically regulated at the colony level.

Conversely, the temporal worker castes allow for a more complex network of inter-member signals, which is made clear in the process of queen production. Any fertilized egg can develop into a queen or a worker; thus, in order for the colony to produce queens, there are mechanisms in the larval stage that affect reproductive caste determination. Specifically, the *nurse* worker temporal caste produces royal jelly (from their hypopharyngeal glands) that causes an epigenetic change in the developing larvae, causing them to switch to the queen developmental program. The *nest worker* temporal caste produces queen cells on the comb in order to allow for the increased growth of queen-larvae. As above, the temporal castes are regulated at the colony level; therefore, typically the nurse and nest worker castes are always present in the colony, enabling them to provide royal jelly and to produce queen cells when required. For this reason, royal jelly and queen cells can be considered as second-order signals on the process of queen production, and conversely, brood food and worker-comb cells can be considered as second-order signals on the process of worker production (Table 2).

In general, the network of inter-member signals in *A. mellifera* results in a much more robust higher order organization compared to colonies of more simple species such as *V. vulgaris*. This is even more evident in the case of the death of the queen. In *V. vulgaris,* colony cohesion rapidly breaks down when the queen dies; workers begin ovipositing (but brood rarely emerge due to multiple eggs being laid in a single comb cell), foraging almost ceases, and cannibalism emerges (Potter, 1964, pp. 50, 62–63). This is because the higher order organization in *V. vulgaris* is completely reliant on the queen; the few second-order signals that affect development and colony cohesiveness derive from the queen. However, in *A. mellifera*, the higher order organization is more resistant to perturbations. If the queen dies, the colony will attempt to replace her. If there is brood present in the nest, *nest workers* will adapt the comb cells of suitable larvae (from fertilized eggs) into queen cells, *nurse workers* will then feed these larvae exclusively with royal jelly and pollen, while the *foragers* continue to forage due to signals from the brood (Pankiw et al., 1998b; Maisonnasse et al., 2010b). During this time the colony remains generally cohesive, due to the complex network of second-order signals that is not solely reliant on the queen.

What do all of these conclusions show? As we have seen, in the case of a eusocial insect species like *A. mellifera*, the colony presents such a high degree of integration that it shows a certain form of individuality. Due to the complex structure of the network of inter-member signals, the colony as a whole emerges as a cohesive organization exerting a set of regulatory controls on the individual bees forming the colony. As a result of these higher level controls, the colony behaves as a reproductive unity, and, in a certain degree, as a physiological and developmental unity.

All this shows a very interesting example of inter-identity, in the sense that it is through the interactions between the different identities of the lower level agents that a new, higher level identity emerges (for a relevant analysis regarding the emergence of multicellular identity in general, see Arnellos, 2018). Interestingly, the identities of the lower level agents, in turn, are affected by the emergent higher level organization insofar as they cannot survive outside the colony. In sum, our case study shows how the conjunction of a set of heterogenous constituent entities forms a complex organization, endowed with its own new identity.

## Author Contributions

All authors contributed equally to the preparation and writing of the manuscript.

### Conflict of Interest

The authors declare that the research was conducted in the absence of any commercial or financial relationships that could be construed as a potential conflict of interest.

## Footnotes

^1^“Colony level” refers to phenomena or processes at the collective level.

^2^This is not to say, of course, that other strategies could not also be used. For example, in the smaller colonies of simpler eusocial species, queen dominance is established and maintained soon after colony formation by physical aggression (Johnson and Linksvayer, 2010).

^3^Our approach is generally based on the autonomous perspective in biology and in particular on the organizational approach, principles, and characteristics of multicellular systems and agents (see Arnellos et al., 2014; Moreno and Mossio, 2015). For a more general argument of the role of hierarchical regulation and control for understanding the constitution of composite (and integrated) organisms, see Bich et al. (2016) and Arnellos and Moreno (2016).

^4^For a more generalized view of evolutionary and physiological notions of biological individuality, as well as an overview of recent debates on the topic, see Pradeu (2016).

^5^The concept of self-organization is also applied to other biological and social phenomena. However, here we are just focusing on the SO approach in the context of eusociality (Detrain and Deneubourg, 2006).

^6^We use the term “queen pheromone mix” as it has been shown that queens produce pheromones from multiple sources—not only their mandibular glands—that act as signals within the colony (Slessor et al., 2005; Maisonnasse et al., 2010a).

^7^Ethyl oleate is also produced by the queen, brood, and other workers. However, it has been argued that only forager-derived ethyl oleate affects the temporal polyethism schedule within workers (Leoncini et al., 2004; Slessor et al., 2005).
